# Chinese students’ access, use and perceptions of ICTs in learning mathematics: findings from an investigation of Shanghai secondary schools

**DOI:** 10.1007/s11858-022-01363-5

**Published:** 2022-04-29

**Authors:** Lianghuo Fan, Jietong Luo, Sicheng Xie, Fangchun Zhu, Shuhui Li

**Affiliations:** 1 East China Normal University, 500 Dongchuan Road, Shanghai, 200241 China; 2 University of Southampton, Southampton, UK

**Keywords:** Chinese mathematics education, ICTs in mathematics education, Learning of mathematics, Shanghai secondary classrooms

## Abstract

Due to rapid social and economic development in China over the last three decades, information and communication technologies (ICTs) have become increasingly available in Chinese classrooms and families. However, there is a lack of research regarding Chinese students’ use of ICTs in mathematics learning. In this study, we examined how Chinese students access, use, and perceive ICTs in learning mathematics. To this end, a conceptual framework for ICTs and the role of ICTs in students’ learning of mathematics was established. Using a mixed-methods approach, we collected data from 223 students in four randomly selected secondary schools in Shanghai through a questionnaire survey, followed by classroom observations and interviews with students and teachers. The results revealed that various ICTs are widely accessible in Shanghai classrooms and students overall have a positive view about the role of ICTs in their mathematics learning, especially in problem solving and in learning geometry. When learning mathematics at home, students used handheld technological devices more frequently than non-portable devices. Furthermore, there were significant differences between different students in terms of school performance levels, grade levels, and genders in their use of ICTs in learning mathematics. In particular, students from high-performing schools had more access to various ICTs but used them less frequently than their peers from ordinary schools. Suggestions and implications of the findings are discussed at the end of the paper.

**Supplementary Information:**

The online version contains supplementary material available at 10.1007/s11858-022-01363-5.

## Introduction

The outstanding performance of Chinese students, particularly Shanghai students, in the Programme for International Student Assessment (PISA) tests in mathematics (OECD, 2010, 2019) has attracted worldwide attention from education policymakers, researchers, and practitioners. Many researchers have studied issues concerning mathematics education in Shanghai, which is the largest city in China and considered to be the nation’s leader in education (UNESCO Institute for Information Technologies in Education et al., 2020), from various perspectives, such as classroom instruction (e.g., Ding et al., 2015), teacher education and professional development (e.g., Tucker, 2014), curriculum and textbooks (e.g., Wang et al., 2017), and social and cultural influences (e.g., Fan et al., 2018). Nevertheless, there is surprisingly little research on Shanghai students’ mathematics learning from a technological perspective.

An earlier related study, a large-scale national survey of nearly 50,000 students in Grade 9 about their mathematics learning, was conducted in China in 1987. The study showed that only 11.4% of the secondary schools were equipped with computers and 12.3% of the students had access to calculators (Tian, 1990). Since then, with rapid social and economic development in China, modern information and communication technologies (ICTs)^1^ have become increasingly available in Chinese classrooms and families. According to the Ministry of Education (MOE), in 2020, 99.7% of primary and secondary schools in China had access to high-speed Internet and 95.2% had multimedia classrooms (MOE, 2020a). By the end of 2020, the coverage rate of household high-speed Internet in China had reached 89.9% (Ministry of Industry and Information Technology, 2021). Against this backdrop, researchers have examined Chinese and Shanghai students’ ICT use in their learning, but most of them focused on students’ general learning (e.g., Luo et al., 2020; OECD, 2019), not particularly in mathematics, and very little is known about how Chinese, let alone Shanghai, students access and use ICTs in their learning of mathematics in school and at home.

Researchers have noted that subject area and learning contexts are important factors influencing students’ use of ICTs (Hawk et al., 2021; Maschietto & Trouche, 2010), and comparisons of different learning contexts may provide valuable insights into designing instructional practices and thus improving the quality of mathematics education (e.g., Kubow & Fossum, 2007). Researchers have also identified that a variety of factors (e.g., school-level factors) may play a role in integrating ICTs into the teaching and learning of mathematics (e.g., Gerick et al., 2017).

In this paper, we present a study regarding the use of ICTs in Shanghai students’ learning of mathematics. More specifically, the study was guided by the following research questions:

1. What ICTs do students in Shanghai have access to, and to what extent do they use ICTs in their learning of mathematics both in school and at home?
2. How do students in Shanghai perceive the role of ICTs in their learning of mathematics?
3. Do differences exist between students in Shanghai in terms of school performance levels, grade levels, and genders in their access to, use of, and perceptions about the role of ICTs in their learning of mathematics?

By addressing these questions, our purpose is to provide research evidence to help understand Chinese students’ learning of mathematics from a technological perspective, and advance the understanding of the role of ICTs in students’ learning of mathematics. In addition to the Chinese context, internationally a number of researchers have examined the issue of how elementary school students (e.g., Selwyn et al., 2009) and post-secondary school students (e.g., Rusli et al., 2020) access, use and perceive ICTs in their learning. We believe this research focus is of importance to the field.

## Related research and conceptual framework

### Teaching and learning of mathematics with ICTs

Integrating technology in the teaching and learning of mathematics is not only an essential topic in the national curriculum in many countries (e.g., Mailizar & Fan, 2020; Ministère de l’Éducation Nationale, 2015; MOE, 1998) but also an active field of research and innovation in recent decades (e.g., De Witte & Rogge, 2014). The rapid development of ICTs in school- and home-affordable forms provides possibilities for enhancing the teaching and learning of mathematics (Triantafyllou & Timcenko, 2013). Research has revealed that, in mathematics education, ICTs make mathematics more authentic by providing opportunities to access real-world data (e.g., Clark-Wilson et al., 2011), make mathematical representations more visual and dynamic (e.g., Vahey et al., 2020), and make mathematical communication and collaboration more convenient (e.g., Geiger et al., 2010). ICTs support students in developing conceptual understanding (e.g., Bos, 2007), improving problem-solving skills (e.g., Granberg & Olsson, 2015), strengthening inquiry-based learning (e.g., Soldano et al., 2019), and promoting students’ interest in mathematics (e.g., Deng et al., 2020). Meanwhile, there also exist concerns about the inappropriate use of ICTs, leading, for example, to the deterioration of students’ competencies in arithmetic skills (e.g., Alhumaid, 2019) and to distractions (Ditzler et al., 2016).

Mainly drawing on the work about mathematics learning and technology by Clark-Wilson et al. (2020) and Verschaffel et al. (2012), in this paper, by looking into *students’ learning of mathematics from a technological perspective*, we refer to students’ acquisition and development of knowledge, skills, and affect related to school mathematics (such as arithmetic, algebra, geometry, probability, and statistics) via a wide range of ICTs, including hardware, software, and the Internet. Our focus is on how students access, use, and perceive ICTs in their learning of mathematics.

According to existing research, the accessibility and frequency of using ICTs in mathematics education have changed over time (e.g., Roschelle et al., 2017). Many researchers have explored various issues concerning using *hardware*, including calculators (e.g., Aldon, 2010) and computers, such as desktops, laptops, and tablets (e.g., Günster & Weigand, 2020). In addition, researchers have looked into multimedia devices, including interactive whiteboards (IWBs), E-readers, and smartphones (e.g., Daher, 2010; Heemskerk et al., 2014). Recently, researchers have also paid attention to handheld technologies, such as graphing calculators, tablets, and smartphones, which make the teachers’ lives easier, improve students’ appreciation, and are free of infrastructural limitations (Trouche & Drijvers, 2010).

Various *software* programs were originally invented for educational purposes, and plenty of them were for mathematics education (Borba et al., 2013; Hillmayr et al., 2020). In fact, drawing on available research, we can classify related software into three types, as follows: (1) learning software—learning resources platform (LRP), learning assessment and management system (LAM), intelligent tutorial system (ITS) or online homework solver or help site, etc.; (2) mathematics software—computer algebra system (CAS), dynamic geometry software/system (DGS), spreadsheets, programming, mathematics games, enrichment such as forum and official account, etc.; and (3) general software—word processor and presentation software, simulation and animation software (simulation), 3D modelling visualization software, online communication and collaboration tools (communication tools), etc. (Bescherer, 2019; Triantafyllou & Timcenko, 2013).

Most countries have invested considerable technological resources in classrooms (e.g., De Witte & Rogge, 2014), and China is no exception. There have been a series of nationwide policy documents published by the MOE emphasizing the use of ICTs in classrooms (e.g., MOE, 1998, 2020a). The Shanghai Mathematics Curriculum Standards also emphasized using calculators, computers, and a purpose-specific ICT platform for mathematics teaching and learning in Shanghai schools, i.e., the Digitization, Information Technology, Modern, and Mathematics Activities (DIMA) platform (Shanghai Municipal Education Commission [SMEC], 2004). Taking into account all the different ICTs as described above, in this study we examined Shanghai students’ use of ICTs by classifying them into different types as shown in Table 1.

**Table 1:** A classification of ICTs for mathematics teaching and learning in Shanghai

Type	Description
Hardware	*Calculator*
	*Computer:* Desktop, laptop, tablet
	*Multimedia device:* IWB, E-reader, smartphone
Software	*Learning:* LRP, LAM, ITS (including online homework solver or help sites)
	*Mathematics:* CAS, DGS, spreadsheet, DIMA, game, enrichment
	*General:* Simulation, communication tool

The issues relating to students’ accessibility and use of ICTs have received attention from researchers worldwide. The TIMSS 2019 study revealed that, with some variation across countries, 68% of eighth graders had mathematics teachers who reported almost no use of computer activities to support learning, and 28–29% of the eighth graders reported working with computers as part of their mathematics lessons (Mullis et al., 2020). Similarly, the International Computer and Information Literacy Study (ICILS) 2018 survey reported that 25% of eighth graders in the 11 participating countries had used computers in most mathematics lessons, and there existed large differences in the accessibility to ICTs across these participating countries (Fraillon et al., 2020). Concerning Chinese students, the PISA 2018 study showed that the percentage of schools with access to the Internet and computers for pedagogical purposes in China (Beijing, Shanghai, Jiangsu, and Zhejiang) was lagging behind other high-performing educational systems in PISA (OECD, 2019). For general pedagogical purposes in school (not particularly for mathematics learning), in France, for example, every 100 students possessed 33.8 computers and 11.7 tablets between 2018 and 2019 (Rosenwald et al., 2020), while in China, the numbers were 15.5 and 1.1, respectively (MOE, 2020b). In particular, in Shanghai, all secondary schools have Internet access and multimedia classrooms since 2015 (MOE, 2015), and every 100 students possessed 36 computers and 5.4 tablets between 2018 and 2019 (MOE, 2020b). However, little is known about Shanghai students’ access to and use of specific hardware and software in their mathematics learning.

Researchers have pointed out several factors that may influence students’ use of ICTs in their mathematics learning. For example, Lowrie et al. (2013) reported that there were significant differences between secondary students of different genders in Australian schools regarding their preferences for digital mathematics games. In a survey of Norwegian seventh to tenth graders concerning their thoughts about ICTs in mathematics, Fuglestad (2006) reported a mixed picture, in that there were significant differences between genders on some attitude questions and between grade levels on other questions. Moreover, the study found very little use of computers in upper secondary schools and not very much in lower grades either. In the same vein, surveying sixth graders in Belgium, Aesaert and van Braak (2015) reported that girls had better technical ICT skills and higher-order ICT competencies than boys. Other studies also found that students’ use of ICTs was positively correlated to (1) school factors (Gerick et al., 2017), (2) ICT self-efficacy (Rohatgi et al., 2016), and (3) the availability of appropriate software (Gil-Flores et al., 2017). On the other hand, based on PISA 2015 data from 44 countries, Hu et al. (2018) used a three-level hierarchical linear model and found that the availability of ICTs in school (positively) and at home (negatively) was associated with students’ academic success. Nevertheless, a research-based systematic understanding of how the school and home settings contribute to students’ use of ICTs in their learning of mathematics is yet to be established.

### Role of ICTs in students’ mathematics learning

Researchers have proposed several frameworks classifying the roles of ICTs in students’ learning of mathematics. Drawing on the conceptualization of Drijvers et al. (2011) about the didactical role of technology, Roschelle et al. (2017) defined four roles of ICTs in mathematics education, as follows:

1. *Doing mathematics*: offload labor that could also be done by hand to tools, such as numeric and symbolic computation.
2. *Practicing problem-solving skills*: organize effective sequencing of tasks, provision of useful feedback, and adaptive pedagogies, such as supporting students online as they do homework.
3. *Developing conceptual understanding*: assist students’ sense-making and understanding of concepts, such as dynamic representations.
4. *Promoting interest-driven learning*: provide informal authentic motivational contexts for mathematics learning, such as designing robots.

Researchers have also argued that ICTs can play a positive role in supporting collaborative learning (Aldon, 2010; Daher, 2010) and inquiry-based learning (Li et al., 2010) in mathematics. According to Borba et al. (2013), the Internet provides on-demand access and support to developing mathematics knowledge, and thus students could collaborate in finishing a mathematics task or sharing ideas without restrictions from geographic locations. Radović et al. (2019) reported that a well-designed communication and collaboration digital tool could support greater connections across and outcomes from home and school mathematics learning for 11- to 14-year-old students in Serbia. The powerful and decisive role of the communication tool in education was manifested during the Covid-19 pandemic in China (Luo et al., 2020). The Shanghai Mathematics Curriculum Standards also highlighted collaborative learning and inquiry-based learning in secondary school mathematics classrooms (SMEC, 2004).

On the other hand, the curriculum and contents of learning are also key factors in conceptualizing the use of ICTs. Fan (2010) reported that many questions in a new series of Singapore secondary mathematics textbooks that are targeted to develop students’ high-level thinking and problem-solving abilities are ICT-embedded, so students can focus more on conceptual understanding, information gathering, logical reasoning, and data analysis instead of tedious calculation, complex algebraic manipulation, or time-consuming drawing in the non-ICT context. In terms of specific mathematics areas, Kaput (1992) examined many examples in arithmetic, geometry, algebra, probability, and statistics and suggested that the appropriate use of ICTs contributes to learning efficiencies. Among these areas, probability and statistics were addressed to a lesser extent, with limited research on this area, even though statistics software such as Fathom and TinkerPlots has been widely used (Drijvers et al., 2009).

The Shanghai Mathematics Curriculum Standards specified the following five mathematics areas: numbers and arithmetic, equations and algebra, functions and analysis, figures and geometry, and data processing, probability, and statistics (SMEC, 2004). There has been little research investigating or comparing the various roles that ICTs play in students’ learning of different content areas in mathematics in a systematic manner.

Based on the above literature review, Table 2 presents the conceptual framework established in this study regarding the role of ICTs in general learning and in area-specific learning in mathematics.

**Table 2:** A conceptual framework about the role of ICTs in mathematics learning

Role	Description
General learning	Develop conceptual understanding
	Practice problem-solving skills
	Promote interest in mathematics
	Strengthen inquiry-based learning
	Enhance communication and collaborative learning
Area-specific learning	Numbers and arithmetic
	Equations and algebra
	Figures and geometry
	Functions and analysis
	Data processing, probability, and statistics

Furthermore, mathematics achievement levels, genders, and grade levels may influence the role that ICTs play in students’ learning of mathematics. Wong et al. (2011) reported that medium-achievement ninth graders in Chinese Taipei enjoyed most interacting with multiple representations provided by a computer-assisted MR Geo, while low-achievement students improved their attitudes towards geometry theorem proving. Again, in Taipei, Lin et al. (2017) found that the designed blended learning environment (Moodle) improved seventh graders’ academic achievement and attitudes towards mathematics, in which male students and high-ability students were more motivated than their counterparts. In a large-scale survey of fifth to eighth graders in the United States, Star et al. (2014) reported that grade levels impacted the role that ICTs played in enhancing students’ motivation in mathematics. Nevertheless, overall, there is inadequate attention paid to the factors influencing the role that ICTs play in students’ learning of mathematics.

In summary, much of the research so far has focused on the technological perspective of mathematics learning in a broader world context, and little research has addressed the context of China, let alone Shanghai. Moreover, the influence of genders, grade levels, and educational settings (school or home) on this issue remains unclear. Furthermore, there has been virtually no investigation into Shanghai students’ use of ICTs. Effort is needed to understand the relationship between mathematics learning in the Chinese context and in the broader world context regarding the accessibility, frequency of use, and role of ICTs. In this regard, this study is intended, for the first time, to present a specific investigation of the availability and use of different ICTs in Shanghai secondary students’ learning of mathematics, as well as the factors influencing the use and role of different ICTs in their learning of mathematics.

## Methods and procedures

This study took place in four randomly selected Shanghai secondary schools. The data were collected through a questionnaire survey, classroom observations, and interviews with students and teachers.

### Research instruments^2^

#### Questionnaire

The questionnaire was designed based on the aforementioned conceptual framework. It consists of four parts. The first part is about students’ demographic information, including gender, month and year of birth, grade level, and their parents’ educational background. The second part focuses on the accessibility of various hardware and software to students at home, the accessibility of software in school^3^, and the frequency of their use in mathematics learning in school and at home. This part also includes fill-in-the-blank questions asking students about the most frequently used and most helpful hardware and software. The third part is about the five roles of ICTs in general, as well as the helpfulness of ICTs in students’ learning of five specific content areas, as described in the conceptual framework above, both on a 4-point Likert scale. Finally, an open-ended question was designed to explore students’ perceptions about the use of ICTs in their learning of mathematics and the reasons behind them.

#### Interview and classroom observation

To triangulate the questionnaire data and gather more in-depth information about how students use particular ICTs and the role that ICTs play in their learning of mathematics, we conducted interviews and classroom observations. The first part of the student interview was a follow-up to the questions in the questionnaire about the most frequently used hardware and software, asking for some typical scenarios in which they used ICTs. The second part was in line with the third part of the questionnaire, asking for examples of how ICTs helped them concerning the five roles of ICTs in general learning and in learning which of the five mathematical areas ICTs were the most helpful. For interviewing teachers, we first asked about the content they taught. We then focused on questions regarding their knowledge about students’ use of ICTs. For example, “From your view, what is the hardware (e.g., computers and tablets) that your students use most frequently when they learn mathematics in school? Could you describe the scenarios in which the hardware is used?”

The classroom observations were aimed to gather evidence about what and how ICTs were used in mathematics classrooms. A specific rubric for classroom observation was designed, including mathematical topics, lesson types, time duration of each mathematical activity, and the use of specific hardware and software in the activity.

To ensure reasonable validity and reliability, a panel of mathematics education researchers was invited to review the instruments. Furthermore, pilot tests with one teacher and two students were conducted separately to refine the questionnaire and interview protocol. Overall, both the teachers and students tested were highly positive about the instruments.

### Data collection and analysis

#### Data collection

Referring to a local education website for the list of high-performing and ordinary schools,^4^ we randomly chose two schools from each category. After obtaining ethics approval to conduct the research, we randomly selected two classes from each school, one from Grade 7 and the other from Grade 8. We chose Grade 7 and 8 for the following reasons: (1) they are free of strong pressure from the high-stakes High School Entrance Examination and thus are more available; and (2) as the content ‘functions and analysis’ is taught in Grade 8, differences in mathematics content may contribute to varied ICT use. In addition, two students randomly chosen from each class and their mathematics teachers participated in the interviews.

We distributed 245 questionnaires to all the participating students and received 223 valid ones, with a response rate of 91.0% and with 113 boys and 110 girls, all aged 12–14. About 15.7% of the students have only one of their parents with an undergraduate degree, 39.6% have both parents with undergraduate degrees, and 24.4% have at least one of their parents with a postgraduate degree; thus, our sample covers a wide range of parent education backgrounds. Table 3 shows the profiles of the participating students.

**Table 3:** Profile of participating students

	High-performing	Ordinary	Subtotal
Grade 7	53 (23.8%)	57 (25.6%)	110 (49.4%)
Grade 8	67 (30.0%)	46 (20.6%)	113 (50.6%)
Subtotal	120 (53.8%)	103 (46.2%)	223 (100.0%)

We recorded and transcribed the interview with 8 teachers and 16 students, in which 10 students were classified as ‘mathematically high-performing’ and 6 students were ‘mathematically ordinary’ according to their teachers. Eight lessons were observed, with five on ‘equations and algebra’, one on ‘figures and geometry’, and two on ‘functions and analysis’. Due to the schools’ scheme of work and the unexpected impact of the Covid-19 pandemic, we were unable to observe more lessons, which is a limitation of the study. For each class, lesson plans, worksheets, and PowerPoint slides were collected. Two observers recorded the use of ICTs in the class, observed independently, then discussed the discrepancies, if any, and finally produced the field notes.

#### Data analysis

Both descriptive and inferential statistics were employed to analyze the data. We calculated each item’s mean and standard deviation in the questionnaire and distinguished several groups of students in terms of genders, school performance levels, and grade levels. To explore factors influencing students’ learning of mathematics in technology-based contexts, we conducted a chi-square test on each item to see if there was a statistically significant difference between students of different groups.

The data about ICT use from the questionnaire, interview transcripts, and classroom observations were used to triangulate the findings. For the interview transcripts, we classified the interviewees’ answers to each question by identifying the types of ICTs they used most frequently or whether they found ICTs helpful in certain aspects or specific mathematics areas.

To maintain anonymity, we referred to the interviewed students as S1 to S16 and interviewed teachers as T1 to T8. To check the interrater reliability, for response to the open-ended question in the questionnaire and all the interview questions, we conducted a Cohen’s kappa test on the coding results between two independent coders. The kappa value for each item’s coding results ranges from 0.83 to 1. The coding results passed the reliability check.

For classroom observations, we looked over the fieldnotes for the ICT use in each classroom activity. Based on the fieldnotes, we summarized students’ accessibility of hardware in school and the percentage of time that ICT was used in class.

## Results and discussion

### The accessibility of ICTs to students and their frequency of use

In the following subsections, we report the results about the accessibility of ICTs to students and frequency of their use, first hardware and then software.

#### Hardware

All classrooms we observed were equipped with IWBs or equivalent touchscreen televisions with in-built computers, overhead, and digital projectors, which was consistent with the aforementioned literature (MOE, 2015). The most frequently used hardware in school was IWBs. In five out of the eight lessons we observed, teachers used IWBs in at least 50.0% of the lesson time for direct instruction and presentation.

During the interview, T7 pointed out that students mainly used tablets in open demonstration classes^5^ instead of regular lessons. Smartphones were the least frequently used hardware in school, with the reason being that the schools have posed regulations that prohibit students from using smartphones during mathematics classes (X. Huang, personal communication, January 16, 2021; M. Jiang, personal communication, January 15, 2021; L. Liu, personal communication, January 23, 2021; F. Yu, personal communication, January 15, 2021).

Table 4 shows the accessibility of hardware to students at home, using the data from the questionnaire. Smartphones exhibited the highest accessibility, with 93.4% of the students having access to smartphones in their mathematics learning at home, while accessibility of E-readers was the lowest (36.2%). There was also a high accessibility of computers (91.4%).

**Table 4:** Students’ access to hardware at home

Hardware	Accessibility		Rank
Calculator	Yes: 171 (83.4%)	No: 34 (16.6%)	3
Computer^a^	Yes: 192 (91.4%)	No: 18 (8.6%)	2
Multimedia device			
E-reader	Yes: 77 (36.2%)	No: 136 (63.8%)	4
Smartphone	Yes: 199 (93.4%)	No: 14 (6.6%)	1

^a^Computer refers to desktop, laptop, or tablet

The results revealed that the hardware most frequently used by the students at home was smartphones, followed by tablets, calculators, desktops, laptops, and E-readers. This result is related to the fact that handheld portable devices, such as smartphones and tablets, provide students with more convenience than desktops and laptops. In addition, the responses of students to one of the fill-in-the-blank questions showed that the most helpful hardware devices were IWBs and smartphones. During the interview, T3 pointed out that compared with computer-based software, mobile apps for smartphones were used more frequently nowadays. Also, 75.0% of the interviewed teachers reported that they sometimes used mobile apps to assign homework to students on weekends, which contributed to students’ frequent use of smartphones or tablets. T2, T5, and T6 also mentioned that students used smartphones or tablets to attend online mathematics lessons at home.

#### Software

The results from the questionnaire data show that Dynamic Geometry Software/System (DGS) was the most accessible software in school (65.4%), followed by the learning resource platform (53.7%) and spreadsheets (48.9%). In school, DGS was the most frequently used software, followed by the learning assessment and management system and learning resource platform. Meanwhile, Computer Algebra System (CAS), games, and DIMA were the three least frequently used software programs. During the interview, S5 felt confused about what exactly DIMA was. Even though DIMA is emphasized in the Shanghai curriculum, it seems to be absent in students’ learning of mathematics. Meanwhile, there are almost no publications about using DIMA in secondary schools and very limited studies on high school teaching practices with DIMA (e.g., Xu, 2019). Thus, we believe that the absence of DIMA is largely related to the lack of essential training.

At home, the communication tool was the most accessible (91.4%) software. The second and third most accessible software programs were the learning resource platform (86.0%) and ITS (81.4%), and the three least accessible were simulation (25.5%), CAS (20.9%), and DIMA (14.2%). In terms of the frequency of use, the communication tool was the most frequently used software, with 69.6% of the students using them more than two times a week, followed by games and the learning resource platform. DIMA, CAS, and simulation were the three least frequently used software programs.

To sum up, in school, Shanghai students nowadays commonly have access to a variety of hardware including IWBs with in-built computers, overhead projectors, and digital projectors, and the most frequently used hardware was IWBs. While at home, the majority of the students had access to smartphones and computers, and they used handheld portable devices in their mathematics learning more frequently than non-portable devices. For software, DGS was most commonly used in school, while the communication tool was most accessible and most frequently used at home. In contrast, DIMA exhibited low accessibility and low frequency of use, indicating a gap between curriculum standards and teaching practice.

### Role of ICTs

In general, the questionnaire data revealed that the majority of students (70.8%) were satisfied with ICT use in mathematics learning, 26.2% thought “it should be increased,” and 3.0% suggested “it should be decreased.” Furthermore, students gave the highest rating to “ICTs help me in learning mathematics overall” (*M* = 3.53), with 98.0% of the students responding with ‘strongly agree’ or ‘agree’, which clearly suggests that ICTs played a highly positive role in students’ learning of mathematics.

Below we report more specific results about the role of ICTs, first in general learning and then in area-specific learning in mathematics.

Regarding students’ general learning of mathematics, the questionnaire data showed that they found ICTs most helpful in problem solving (*M* = 3.46), followed by inquiry-based learning (*M* = 3.42), collaborative learning (*M* = 3.35), conceptual understanding (*M* = 3.34), and finally interest in mathematics (*M* = 3.33). There is no doubt from the results that, overall, the students have a positive view about the role of ICTs in their general learning of mathematics. During the interviews, the students gave many examples of how ICTs helped them in problem solving, and half of them mentioned that they would search for relevant information online to solve mathematics problems. Not only would they search for the right answers to challenging problems, but they would also search for related mathematics concepts. S1, S3, and S8 also pointed out that the Geometer’s Sketchpad (GSP) could help them solve geometry problems. S6 posed problems in mathematics online forums seeking help.

**Fig. 1:**
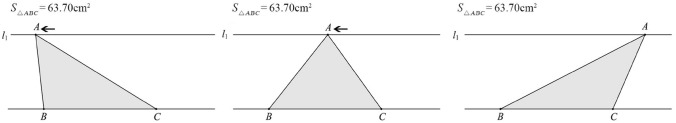
Illustrations produced by GSP

The following is an example given by S7 about conceptual understanding and inquiry-based activities exploring the theorem ‘triangles with the same base and equal heights have equal areas’:

It is difficult to understand this theorem without ICTs. This is where ICTs can make a difference (see Fig. 1, for example). Using GSP, you draw a line $l_{1}$ parallel to the base $BC$ across the vertex $A$, and then you can drag the vertex $A$ along the line $l_{1}$ and see that the area of triangle $ABC$ stays the same… ICTs help me a lot in exploring more approaches to solve the same problem. And thus, I could save more time by choosing the most efficient approach.

In the interviews, nine students pointed out that when they felt confused about particular concepts, they would search them online or study again through videos. S1, S2 and S4 also said that communication tools allowed them to discuss challenging problems with their classmates after class freely.

On average, students also agreed that ICTs promoted their interest in mathematics, with a mean value of the rating being over 3. During the interview, S10 and S13 mentioned that “some dynamic animations make mathematics more vivid and interesting,” which we think explains that ICTs can make the learning of specific mathematical topics more interesting to students, even though the students in Shanghai may already have had strong interest in mathematics. In this regard, it was also a bit surprising to us that ‘promoting interest in mathematics’ received the lowest rating on the questionnaire. During the interview, S14 argued, “I am interested in mathematics with or without ICTs,” which might be related to the fact that Shanghai students’ interest in mathematics was relatively high (National Assessment Center for Education Quality, 2018); hence, the role of ICTs in promoting students’ learning interest is positive, but not as large as one might have expected, in the Shanghai context. In this connection, T8 spoke as follows:

Rather than enhancing students’ interest in mathematics, ICTs indeed help my students build their sense of accomplishment. For example, students are motivated and even feel the magic happening when I use GSP in class. I would suggest my students explore GSP after class. When they succeeded in designing figures and exploring new concepts with GSP by themselves, they would feel a great sense of accomplishment.

In terms of area-specific learning, the questionnaire data showed that students found ICTs most helpful in their learning of figures and geometry (*M* = 3.46), followed by functions and analysis (*M* = 3.42), data processing, probability, and statistics (*M* = 3.30), numbers and arithmetic (*M* = 3.26), and finally equations and algebra (*M* = 3.18). Again, the results revealed that the students had a rather positive view about the role of ICTs in their learning of all the specific areas in mathematics. During the interview, many examples were provided from both students and teachers to illustrate how ICTs played an important role in learning figures and geometry as well as functions and analysis. For example, 10 students and 4 teachers mentioned that DGS was very helpful in drawing and showing the dynamic movements of figures, such as rotation and translation, which are challenging to be learned without ICTs. Also, T8 mentioned, “Students usually mix up axially symmetric figures and two figures that are symmetric about a line. GSP is very helpful in showing the dynamic movements and then students can easily grasp the difference between these two ideas.” Regarding functions and analysis, T2 said, “With ICTs, I can present various representations [of functions] simultaneously, which makes learning functions more vivid and intuitive.” In comparison, the role of ICTs in learning equations and algebra was not as helpful as in learning other areas by students, which is consistent with the aforementioned finding that Shanghai students rarely used software (e.g., CAS) when learning equations and algebra in school and at home. In addition, the low frequency of ICT use in algebra classes may contribute to students’ perception that ICTs are not that helpful in learning equations and algebra.

In the open-ended question, some students expressed their concerns about the improper use of ICTs, which could hinder their mathematics learning. For example, three students criticized online homework solvers, as some of their classmates directly searched for solutions online instead of solving problems by themselves. As we noticed from the classroom observation, ICT use in school was mostly teachers’ presenting content to students, which, to some degree, reduced the potential that ICTs can contribute to students’ mathematics learning. For example, in the lesson we observed on ‘figures and geometry’, T1 used IWBs to demonstrate several properties of parallel lines, explain worked-out examples in textbooks, and show a student’s answer to an exercise problem, whereas students’ actual use of ICTs in mathematics classrooms was still limited.

In short, ICTs played a positive role in Shanghai students’ learning of mathematics and were considered most helpful in students’ problem solving and least helpful in promoting interest in mathematics.

### Differences in ICT use between different students

The chi-square tests showed that there existed various statistically significant differences between different students in terms of school performance levels, grade levels, and genders in their access to, use of, and perceptions about the role of ICTs in their learning of mathematics, as given in Table 5.

**Table 5:** Significant differences between different students in terms of access to, frequency of using, and role of ICTs in their learning of mathematics

	Type of ICTs	Accessibility (at home)	Frequency of use		Role of ICTs
			In school	At home	
*Hardware*					*General learning*
Calculator		SPL: 11.670***		GL: 16.253**	Conceptual understanding—SPL: 5.825*
Computer	Desktop	SPL: 7.119**	SPL: 24.284***; GL: 24.410***	G: 10.779*	Collaborative learning—SPL: 4.337*; G: 5.550*
	Laptop	SPL: 7.086**		SPL: 8.275*	
	Tablet	SPL: 6.581*			
Multimedia devices	E-reader	GL: 4.359*			
	Smartphone	G: 6.966**			
*Software*					*Area-specific learning*
Learning	Learning resource platform		SPL: 25.538***; GL: 15.402**		Numbers and arithmetic—SPL: 5.718*
	Learning assessment and management system	GL: 31.666***	SPL: 14.420**		
Mathematics	Dynamic geometry software/system	SPL: 10.609**	GL: 10.229*		
	Spreadsheet	SPL: 31.701***			
	Computer algebra system	SPL: 5.409*			
	DIMA	SPL: 5.760*			
	Enrichment	SPL: 4.536*; GL: 4.703*			
General	Communication tool	GL: 7.071**		GL: 11.107*	

DIMA = Digitization, Information Technology, Modern, and Mathematics Activities platform; SPL = school performance level, GL = grade level, G = gender

*p < 0.05, **p < 0.05, ***p < 0.001

#### School performance level

As shown in Table 5, students from high-performing schools had significantly more access to all the hardware devices at home at the 0.05 level except for E-readers and smartphones, which may be related to different social-economic statuses. However, students from high-performing schools used desktops in school and laptops at home significantly less frequently than students from ordinary schools. Regarding software, high-performing school students had significantly more access at the 0.05 level to software (except for LRP, LAM, ITS, games, and communication tools) than ordinary school students at home, while their LRP use in school and LAM use at home were significantly less frequent than that of their counterparts. The differences in use may be related to the role of ICTs in their conceptual understanding.

In terms of the general role of ICTs in mathematics learning, there was no significant difference between students from different school performance levels with regard to the role of ICTs in mathematics learning, except for conceptual understanding and collaborative learning. In fact, the questionnaire showed that 96.7% of the students in ordinary schools held a positive view about the role of ICTs in their learning for conceptual understanding, while only 87.3% of the students from high-performing schools held that view. Similarly, the percentage of students in ordinary schools holding the view of a positive role of ICTs in their collaborative learning was higher than that of their peers from high-performing schools (96.7% vs. 89.0%). In other words, students from ordinary schools valued ICTs as significantly more helpful in conceptual understanding and collaborative learning.

The interview data revealed that seven students from ordinary schools tended to discuss problems via communication tools. In contrast, four students from high-performing schools pointed out that they hardly used ICTs to learn mathematics collaboratively. The open-ended question also exhibited distinct views on the role of ICTs in conceptual understanding, where one student from a high-performing school said that “mathematics learning requires one’s own deep understanding to a great extent, perhaps ICTs can offer some aids, but there is no need to have too much.” Another student from an ordinary school thought that “ICTs help us understand mathematics concepts better and acquire mathematical knowledge more quickly than without ICTs.”

For the role of ICTs in the learning of specific topic areas, there was no significant difference between students from different school performance levels, except for numbers and arithmetic. Students from ordinary schools regarded ICTs as more helpful in learning numbers and arithmetic than their counterparts. About 22.3% of the students from high-performing schools (strongly) disagreed that ICTs helped them in learning numbers and arithmetic, while only 9.8% of those from ordinary schools held the same view.

#### Grade level

The eighth graders had significantly more access to E-readers and used desktops in school and calculators at home significantly more frequently than the seventh graders. The different usage of calculators was probably because the eighth graders needed to do more complicated calculations in solving real-life problems related to functions than the seventh graders.

For software, the eighth graders had significantly more access to LAM, enrichment, and communication tools than the seventh graders. In school, the eighth graders used LRP and DGS significantly more frequently than the seventh graders. Also, 53.1% of the eighth graders used DGS at least three times a week, but only 27.5% of the seventh graders did so. As we asked the interviewed teachers and went through the textbooks, we found that this result might be related to the fact that eighth graders were learning geometry-related content (i.e., content regarding figures and geometry and functions and analysis) in that semester. Since there exists an interaction between grade levels and mathematical subject content, we could say that the grade differences in ICT use in school are largely due to the content differences in mathematics learning between the two grades. At home, the eighth graders used communication tools significantly more frequently than the seventh graders in learning mathematics. Moreover, there was no significant difference between grade levels in the role of ICTs in general mathematics learning.

#### Gender

There was no statistically significant difference in the accessibility and usage of hardware and software between the two genders, except that girls had significantly more access to smartphones than boys at home and used desktops significantly less frequently than boys at home.

Also, there was no significant difference between the genders, except for collaborative learning. Overall, girls evaluated ICTs as more helpful in collaborative learning than did boys, as the percentage of girls who strongly agreed or agreed about the positive role of ICTs in this aspect was higher than that of boys (97.0% > 88.2%). According to the interviews, seven girls preferred to discuss mathematics tasks through communication tools, whereas boys tended to do collaborative learning face-to-face. It would be interesting to know the reasons for such differences between students of different genders, an issue worth further study.

## Summary and concluding remarks

This study had the aim of investigating how Shanghai students access, use, and perceive ICTs concerning their learning of mathematics. By drawing on research literature and taking Shanghai educational contexts into account, for the study we established a conceptual framework concerning ICTs and the role of ICTs in students’ learning of mathematics. The data were collected using a mixed-methods approach from 223 students in four randomly selected secondary schools, through a questionnaire survey, classroom observations, and interviews. From the findings and discussion above, the following conclusions can be drawn.

First, various ICTs are widely accessible in Shanghai students’ learning of mathematics. In other words, Shanghai students have largely adequate learning opportunities to use ICTs in school and at home. In school, virtually all the students in Shanghai have access to a technology-based environment with computers, IWBs, and the Internet, though they have no access to smartphones due to school rules. At home, a large majority (more than 90%) of the students have access to smartphones and computers, and most (83%) to calculators and some (36%) to E-readers.

Second, Shanghai students’ actual use of ICTs in their learning of mathematics in school and at home is rather varied, with limited use in school but diverse use at home. While at home they used handheld portable devices more frequently than non-portable devices, in school their use of ICTs involved mostly watching teachers’ presentations on IWBs, without providing many learning opportunities for students to actively use ICTs based on their own unique situations, which is similar to the case reported in the study of Yarbro et al. (2016) that US mathematics teachers (Grades 7 to 10) used the digital teaching strategy more frequently for direct instruction of content. In this regard, we think, as Eickelmann et al. (2017) claimed, apart from accessibility and frequency of using ICTs, the issues of ‘how to effectively use ICTs’ merit more attention in future research, as a high frequency of using specific ICTs does not always mean active or creative use for optimal learning.

Third, Shanghai students overall have a highly positive view of ICTs in their mathematics learning. Nevertheless, there exist differences regarding the role of ICTs in different aspects of learning mathematics. ICTs are considered most helpful in students’ learning of problem solving and doing inquiry-based activities, which is consistent with previous studies reporting the supporting role of ICTs in problem solving (e.g., Granberg & Olsson, 2015). The role of ICTs in promoting students’ interest in learning mathematics is also perceived positively, but not as much as in other aspects such as conceptual understanding, inquiry-based learning, problem solving, and collaborative learning. The reason appears related to the fact that Shanghai students already have had strong interest in mathematics and thus what ICTs can contribute in this aspect is limited.

Fourth, regarding specific content areas, ICTs play the most facilitating role in students’ learning of figures and geometry, followed by functions and analysis and the least in equations and algebra. This is largely related to the fact that Shanghai students use DGS with a high frequency, while using software related to algebra (e.g., CAS) with a low frequency (see also OECD, 2020).

Fifth, there exist significant differences between different students in terms of school performance levels, grade levels, and genders in their use of ICTs in the learning of mathematics. Students from high-performing schools have more access to various ICTs but use ICTs less frequently than their peers from ordinary schools. This result is partly related to the fact that students from ordinary schools hold more positive perceptions of the role of ICTs in their conceptual understanding, collaborative learning, and learning numbers and arithmetic than do their peers from high-performing schools. In addition, compared with students from ordinary schools, students from high-performing schools are generally high-performing with better mathematics backgrounds and hence rely less on using ICTs in their learning of mathematics. In this regard, previous international comparative studies have revealed that the frequency of using ICTs is not always positively correlated with students’ mathematics achievement (e.g., Eickelmann et al., 2017; Hu et al., 2018; Odell et al., 2020), and thus ICTs might not be the main factor determining students’ mathematics achievement. Further studies should be conducted to examine the relationships between students’ mathematics achievement and ICT use in their learning of mathematics.

Regarding grade levels, the study found that students at a higher grade level (Grade 8) generally use ICTs, such as calculators and communication tools at home and DGS in school, more frequently than students at a lower grade level (Grade 7). We think this result is largely related to the content they learn in the different grades since the content ‘functions and analysis’, which contains rich learning opportunities for utilizing DGS and calculators, is taught in Grade 8. In addition, as some earlier studies (e.g., Star et al., 2014) noted, such grade differences in using ICTs could be related to students’ developmental levels. Further research is needed to explore what other reasons (e.g., curriculum sequencing) might cause such a difference.

Regarding the gender difference, female students considered ICTs more helpful in collaborative learning than male students, and no statistically significant difference was found between different genders in the frequency of using most ICTs (except for desktops at home) or in their role in promoting interest in mathematics. Interestingly, earlier studies found that boys expressed more positive views towards ICT use in their learning of mathematics than girls (Barkatsas et al., 2009), and girls were less confident and competent than boys in their use of ICTs for learning mathematics (Tan, 2015). We think the gender differences in the perceptions regarding the role of ICTs in collaborative learning reported in our study might be related to their attitudes towards collaborative learning. In fact, as mentioned earlier, the interview data in this study showed that more girls preferred to do collaborative learning online using ICTs, while more boys preferred to do collaborative learning face-to-face. Nevertheless, we think it calls for further research to examine issues more specifically concerning the gender difference in using ICTs for mathematics learning, and moreover, how different ICT tools and educational contexts provide different learning opportunities concerning the use of ICTs to promote students of different genders in their learning of mathematics.

Finally, we should point out that, although this study was based on the Shanghai educational settings, some results and general observations revealed in the study, such as the gap between intended use in curriculum and actual use of ICTs in practice, and the learning opportunities ICTs provided or the role of ICTs for different groups of students in learning different contents in mathematics, may have general implications that go beyond the Shanghai context. In this regard, further research is warranted to advance our understanding of what and how ICTs play a role in students’ learning of mathematics in different educational settings with different economic, cultural, and social contexts.

## Supplementary Information

Below is the link to the electronic supplementary material.

Supplementary file1 (DOCX 70 kb)

Supplementary file2 (DOCX 56 kb)

Supplementary file3 (DOCX 75 kb)

## Footnotes

1 In this study, we use ICTs in the plural to refer to a collection of information and communication technologies.

2 The English version of the questionnaire, interview protocol, and classroom observation rubrics can be seen in the Electronic Supplementary Material.

3 The data of the accessibility of hardware to students were collected through classroom observations.

4 http://edu.online.sh.cn/education/gb/content/2019-11/19/content_9439937.htm.

5 An open demonstration class means that the class is open for others to observe, often for teacher professional development.
