# Supplementary material for: Chinese students’ access, use and perceptions of ICTs in learning mathematics: findings from an investigation of Shanghai secondary schools
Source: ZDM. 2022 Apr 29;54(3):611–24. doi: 10.1007/s11858-022-01363-5 (PMC9052737; doi:10.1007/s11858-022-01363-5)
Supplement: Supplementary file 1 — Supplementary file1 (DOCX 70 kb) [file 11858_2022_1363_MOESM1_ESM.docx]

**Survey for Shanghai secondary students’ use of ICTs in mathematics learning**

**Name (Optional) __________ School (Optional) ________________ Date ___________**

**I. Personal Information (Please tick the box)**

1. Gender: □Male □Female
2. Month and year of birth: (Year) (Month)
3. Grade: □Seven □Eight
4. Your father’s educational background:

□High school/vocational school diploma or lower □Undergraduate □Graduate (Master/Doctor)

1. Your mother’s educational background:

□High school/vocational school diploma or lower □Undergraduate □Graduate (Master/Doctor)

**II. The following questions are about whether you have access to modern Information and Communication Technologies (ICTs, including hardware and software), and the frequency of using these ICTs in mathematics learning (starting from Grade 6). Please answer the questions based on your own situation. If you are not clear about the meaning of some terms, you can ask your teacher, or the researcher responsible for this study, Dr. Li (Tel: XXXXXXXXXXX).**

Please circle:

① For the “Accessibility” of ICT, please circle the option “Yes” or “No”, **if you choose “No”, you do not need to circle the “Frequency of use”**;

② For the “Frequency of use”, the numbers stand for: 4 – always (at least 5 times a week); 3 – usually (3-4 times a week); 2 – sometimes (once or twice a week); 1 – never.

**1. Please answer the following questions about whether you have access to hardware and the frequency of using them in mathematics learning:**

| **ICT** | | **In school** | | | | **At home** | | | | |
| --- | --- | --- | --- | --- | --- | --- | --- | --- | --- | --- |
|  |  | **Frequency of use (in mathematics learning)** | | | | **Accessibility** | **Frequency of use (in mathematics learning)** | | | |
|  |  | ***Always*** | ***Usually*** | ***Sometimes*** | ***Never*** |  | ***Always*** | ***Usually*** | ***Sometimes*** | ***Never*** |
| a. Calculator | | 4 | 3 | 2 | 1 | No Yes | 4 | 3 | 2 | 1 |
| b. Computer | b1. Desktop | 4 | 3 | 2 | 1 | No Yes | 4 | 3 | 2 | 1 |
|  | b2. Laptop | 4 | 3 | 2 | 1 | No Yes | 4 | 3 | 2 | 1 |
|  | b3. Tablet | 4 | 3 | 2 | 1 | No Yes | 4 | 3 | 2 | 1 |
| c. Multimedia device | c1. E-reader | 4 | 3 | 2 | 1 | No Yes | 4 | 3 | 2 | 1 |
|  | c2. Interactive whiteboard (touchscreen television) | 4 | 3 | 2 | 1 | No Yes | 4 | 3 | 2 | 1 |
|  | c3. Smartphone | 4 | 3 | 2 | 1 | No Yes | 4 | 3 | 2 | 1 |
| d. Others | d1. Digital projector | 4 | 3 | 2 | 1 | No Yes | 4 | 3 | 2 | 1 |
|  | d2. Overhead projector | 4 | 3 | 2 | 1 | No Yes | 4 | 3 | 2 | 1 |
|  | d3. Please specify: | 4 | 3 | 2 | 1 | No Yes | 4 | 3 | 2 | 1 |
|  | d4. Please specify: | 4 | 3 | 2 | 1 | No Yes | 4 | 3 | 2 | 1 |

**Please fill in the blanks:**

e. Among the abovementioned hardware, the **most frequently used** one(s) in your mathematics learning during this semester is/are .

f. Among the abovementioned hardware, the one(s) that you find **most helpful** in your mathematics learning during this semester is/are .

**2. Please answer the following questions about whether you have access to software and the frequency of using them in mathematics learning:**

| **ICT** | **In School** | | | | | **At home** | | | | |
| --- | --- | --- | --- | --- | --- | --- | --- | --- | --- | --- |
|  | **Accessibility** | **Frequency of use (in mathematics learning)** | | | | **Accessibility** | **Frequency of use (in mathematics learning)** | | | |
|  |  | ***Always*** | ***Usually*** | ***Sometimes*** | ***Never*** |  | ***Always*** | ***Usually*** | ***Sometimes*** | ***Never*** |
| **Software for learning:** | | | | | | | | | | |
| a. Learning resource platform (e.g., XueKe, Tencent Class) | No Yes | 4 | 3 | 2 | 1 | No Yes | 4 | 3 | 2 | 1 |
| b. Learning assessment and management platform (e.g., K12 keketong, Jyeoo) | No Yes | 4 | 3 | 2 | 1 | No Yes | 4 | 3 | 2 | 1 |
| c. Intelligent tutoring system or online homework solver or help site (e.g., Zuoyebang, Yuansouti) | No Yes | 4 | 3 | 2 | 1 | No Yes | 4 | 3 | 2 | 1 |
| **Software for learning mathematics:** | | | | | | | | | | |
| d. Dynamic Geometry System (e.g., Super Sketchpad, Geometer’s Sketchpad, GeoGebra, SketchUp) | No Yes | 4 | 3 | 2 | 1 | No Yes | 4 | 3 | 2 | 1 |
| e. Statistical software (e.g., Excel, SPSS) | No Yes | 4 | 3 | 2 | 1 | No Yes | 4 | 3 | 2 | 1 |
| f. Computer Algebra System (CAS) (e.g., Maple, Maxima) | No Yes | 4 | 3 | 2 | 1 | No Yes | 4 | 3 | 2 | 1 |
| g. DIMA | No Yes | 4 | 3 | 2 | 1 | No Yes | 4 | 3 | 2 | 1 |
| h. Mathematics game (e.g., 2048, Sudoku) | No Yes | 4 | 3 | 2 | 1 | No Yes | 4 | 3 | 2 | 1 |
| i. Mathematics enrichment (e.g., Mathematics forums, Video platforms, Official accounts) | No Yes | 4 | 3 | 2 | 1 | No Yes | 4 | 3 | 2 | 1 |
| **Others:** | | | | | | | | | | |
| j. Simulation software (e.g., Flash, Fathom) | No Yes | 4 | 3 | 2 | 1 | No Yes | 4 | 3 | 2 | 1 |
| k. Online communication and collaboration tool (e.g., DingTalk, WeChat, QQ) | No Yes | 4 | 3 | 2 | 1 | No Yes | 4 | 3 | 2 | 1 |
| l. Others (Please specify): | No Yes | 4 | 3 | 2 | 1 | No Yes | 4 | 3 | 2 | 1 |
| m. Others (Please specify): | No Yes | 4 | 3 | 2 | 1 | No Yes | 4 | 3 | 2 | 1 |

**Please fill in the blanks:**

n. Among the abovementioned software, the **most frequently used** one(s) in your mathematics learning during this semester is/are .

o. Among the abovementioned software, the one(s) that you find **most helpful** in your mathematics learning during this semester is/are .

**III. In your learning of mathematics, do ICTs help your mathematics learning? Please answer according to your own situation.**

**3. How does the use of ICTs help your mathematics learning?**

[Please circle: 4 – Strongly agree; 3 – Agree; 2 – Disagree; 1 – Strongly disagree; N/A – Not applicable]

| **Using ICTs can help me in learning mathematics** | ***Strongly agree*** | ***Agree*** | ***Disagree*** | ***Strongly disagree*** | ***Not applicable*** |
| --- | --- | --- | --- | --- | --- |
| a. Overall, using ICTs can help me in learning mathematics. | 4 | 3 | 2 | 1 | N/A |
| b. Specifically, using ICTs can help me in the learning of mathematics regarding the following aspects： | | | | | |
| (1) Using ICTs can help me develop conceptual understanding. | 4 | 3 | 2 | 1 | N/A |
| (2) Using ICTs can help me practice problem-solving skills. | 4 | 3 | 2 | 1 | N/A |
| (3) Using ICTs can help me strengthen inquiry-based learning. | 4 | 3 | 2 | 1 | N/A |
| (4) Using ICTs can help promote my interest in mathematics. | 4 | 3 | 2 | 1 | N/A |
| (5) Using ICTs can help me enhance communication and collaborative learning with my classmates. | 4 | 3 | 2 | 1 | N/A |
| (6) Using ICTs can help me in other aspects (Please specify): | 4 | 3 | 2 | 1 | N/A |
| (7) Using ICTs can help me in other aspects (Please specify): | 4 | 3 | 2 | 1 | N/A |

**4. How useful are ICTs in your learning of the following five mathematics areas (fields)?**

[Please circle: 4 – Very useful; 3 – Useful; 2 – Not very useful; 1 – Not useful; N/A – Not sure]

| **Mathematics areas** | ***Very useful*** | ***Useful*** | ***Not very useful*** | ***Not useful*** | ***Not sure*** |
| --- | --- | --- | --- | --- | --- |
| a. Numbers and arithmetic (e.g., rational numbers) | 4 | 3 | 2 | 1 | N/A |
| b. Equations and algebra (e.g., equations) | 4 | 3 | 2 | 1 | N/A |
| c. Figures and geometry (e.g., triangles, rectangular) | 4 | 3 | 2 | 1 | N/A |
| d. Functions and analysis* (e.g., functions) | 4 | 3 | 2 | 1 | N/A |
| e. Data processing, probability and statistics (e.g., random events) | 4 | 3 | 2 | 1 | N/A |

*For seventh graders, please ignore this item, as you haven’t learned this area yet.

**IV. Open-ended question**

**In your learning of mathematics**, do you think the current use of ICT **should be increased/should be decreased/ is just the right amount**? Why?
