# Supplementary material for: Chinese students’ access, use and perceptions of ICTs in learning mathematics: findings from an investigation of Shanghai secondary schools
Source: ZDM. 2022 Apr 29;54(3):611–24. doi: 10.1007/s11858-022-01363-5 (PMC9052737; doi:10.1007/s11858-022-01363-5)
Supplement: Supplementary file 2 — Supplementary file2 (DOCX 56 kb) [file 11858_2022_1363_MOESM2_ESM.docx]

**Outlines of interview**

**Questions for Students**

1. What is the hardware (e.g., computers and tablets) that you use most frequently when you learn mathematics in school? Could you describe one of the scenarios in which this type of hardware is used?

2. What is the hardware (e.g., computers and tablets) that you use most frequently when you learn mathematics at home? Could you describe one of the scenarios in which this type of hardware is used?

3. What is the software (e.g., Geometer's Sketchpad and mathematics games) that you use most frequently when you learn mathematics in school? Could you describe one of the scenarios in which this type of software is used?

4. What is the software (e.g., Geometer's Sketchpad and mathematics games) that you use most frequently when you learn mathematics at home? Could you describe one of the scenarios in which this type of software is used?

5. Do you think ICTs can help you develop conceptual understanding? Please give an example.

6. Do you think ICTs can help you practice problem-solving skills? Please give an example.

7. Do you think ICTs can help you strengthen inquiry-based learning? Please give an example.

8. Do you think ICTs can promote your interest in mathematics? Please give an example.

9. Do you think ICTs can help you enhance communication and collaborative learning with your classmates? Please give an example.

10. In learning which of the five mathematical areas (i.e., numbers and arithmetic; equations and algebra; figures and geometry; functions and analysis; data processing, probability and statistics) do you think ICTs are the most useful? Please give an example.

**Questions for Teachers**

Perquisite question: Which area(s) of mathematics have you taught during this semester (i.e., numbers and arithmetic; equations and algebra; figures and geometry; functions and analysis; data processing, probability and statistics)?

1. From your view, what is the hardware (e.g., computers and tablets) that your students use most frequently when they learn mathematics in school? Could you describe one of the scenarios in which this type of hardware is used?

2. From your view, what is the hardware (e.g., computers and tablets) that your students use most frequently when they learn mathematics at home? Could you describe one of the scenarios in which this type of hardware is used?

3. From your view, what is the software (e.g., Geometer's Sketchpad and mathematics games) that your students use most frequently when they learn mathematics in school? Could you describe one of the scenarios in which this type of software is used?

4. From your view, what is the software (e.g., Geometer's Sketchpad and mathematics games) that your students use most frequently when they learn mathematics at home? Could you describe one of the scenarios in which this type of software is used?

5. From your view, do you think ICTs can help your students develop their conceptual understanding? Please give an example.

6. From your view, do you think ICTs can help your students practice problem-solving skills? Please give an example.

7. From your view, do you think ICTs can help your students strengthen inquiry-based learning? Please give an example.

8. From your view, do you think ICTs can help your students promote their interest in mathematics? Please give an example.

9. From your view, do you think ICTs can help your students enhance their communication and collaborative learning? Please give an example.

10. From your view, in learning which of the five mathematical areas (i.e., numbers and arithmetic; equations and algebra; figures and geometry; functions and analysis; data processing, probability and statistics) do you think ICTs are the most useful for your students? Please give an example.
