# Supplementary material for: Chinese students’ access, use and perceptions of ICTs in learning mathematics: findings from an investigation of Shanghai secondary schools
Source: ZDM. 2022 Apr 29;54(3):611–24. doi: 10.1007/s11858-022-01363-5 (PMC9052737; doi:10.1007/s11858-022-01363-5)
Supplement: Supplementary file 3 — Supplementary file3 (DOCX 75 kb) [file 11858_2022_1363_MOESM3_ESM.docx]

**Classroom Observation Record**

**School: Grade: Observer:**

**Date and time: Type of the lesson: Topic:**

| **Activity** | **Duration** | **ICT(s) used** | **Time spent on using ICT(s)** | **Brief description of ICT(s) use** |
| --- | --- | --- | --- | --- |
| Activity 1 |  | **Hardware:** |  |  |
|  |  | **Software:** |  |  |
| Activity 2 |  | **Hardware:** |  |  |
|  |  | **Software:** |  |  |
| Activity 3 |  | **Hardware:** |  |  |
|  |  | **Software:** |  |  |
| Activity 4 |  | **Hardware:** |  |  |
|  |  | **Software:** |  |  |
| Activity 5 |  | **Hardware:** |  |  |
|  |  | **Software:** |  |  |
| … |  |  |  |  |

**Notes:**

**Hardware:**

Calculator [Including graphing calculator];

Computer [Desktop, Laptop, Tablet];

Multimedia device [E-reader, Interactive whiteboard (touchscreen television), Smartphone];

Others [Digital projector, Overhead projector, Other hardware].

**Software:**

For learning [Learning resource platform (e.g., XueKe, Tencent Class), learning assessment and management system (e.g., K12 keketong, Jyeoo), intelligent tutoring system or online homework solver or help site (e.g., Zuoyebang, Yuansouti)];

For learning mathematics [Dynamic Geometry System (e.g., Super Sketchpad, Geometer’s Sketchpad, GeoGebra, SketchUp), Statistical software (e.g., Excel, SPSS), Computer Algebra System (CAS) (e.g., Maple, Maxima), DIMA, Mathematics game (e.g., 2048, Sudoku), Mathematics enrichment (e.g., Mathematics forums, Official accounts)];

General [Simulation software (e.g., Flash, Fathom), Online communication and collaboration tool (e.g., DingTalk, WeChat, QQ), Other software].
